# A case report: high dose melphalan as a conditioning regimen for multiple myeloma induces sinus arrest

**DOI:** 10.1186/s40959-020-0059-0

**Published:** 2020-03-13

**Authors:** Liang-Liang Ma, Ying Liu, Si-Xun Jia, Hai-Chen Lv, Mei-Yun Fang, Yun-Long Xia

**Affiliations:** 1 Department of Hematology, the First Affiliated Hospital of Dalian Medical University, Dalian, China; 2 Department of Cardiology, the First Affiliated Hospital of Dalian Medical University, 222 Zhongshan Road, Dalian, 116021 Liaoning China; 3 Department of Hematology, Affiliated Zhongshan Hospital of Dalian University, 6 Jiefang Road, Dalian, 116001 Liaoning China

**Keywords:** Melphalan, Multiple myeloma, Autologous stem cell transplantation, Sinus arrest, Arrhythmia

## Abstract

High dose melphalan is commonly used as a conditioning regimen for autologous stem cell transplantation in multiple myeloma. There are reports of adverse cardiac events with melphalan manifested by supraventricular tachycardia and atrial fibrillation. Here, we report a rare case of a 58 year old female with multiple myeloma, who developed sinus arrest after autologous stem cell transplantation using high dose melphalan as a conditioning regimen. It was severe and rare, therefore, monitoring for cardiac toxicity in patients receiving high-dose melphalan is mandatory.

## Background

With advances made in medical treatment and autologous stem cell transplantation (ASCT), clinical response and survival rates of multiple myeloma (MM) have improved. High dose melphalan (200 mg/m^2^) is the international standard for conditioning before ASCT for MM [1, 2]. Cardiac toxicity in the form of supraventricular tachycardia (SVT) and atrial fibrillation (AF) are common after high dose melphalan therapy [3–5]. Melphalan is the most arrhythmogenic agent, and is associated with SVT in 11% of patients, especially in elderly patients with cardiovascular comorbidities [4]. We presented a rare case of the patient with MM developed sever cardiotoxic events (sinus arrest and ass attack) secondary to high dose melphalan who was saved by implanting permanent heart pacemaker. Monitoring for cardiac toxicity in patients receiving high-dose melphalan is mandatory.

**Fig. 1:**
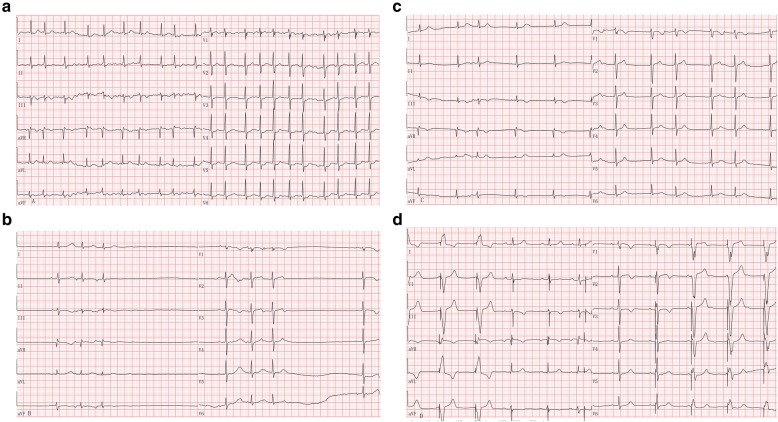
ECG tracings of the patients (25 mm/s,10 mm/mV). **a** Atrial fibrillation (sinus:131 bpm) (d + 9) **b** Premature onset,sinus arrest or sinus block (d + 18). Sudden chest tightness, shortness of breath, followed by ass attack was occurred. **c** Borderline escape rhythm with sinus capture (d + 22). A temporary heart pacemaker had been placed for 3 days. **d** Spontaneous rhythm and pacemaker heart rate alternately appear (d + 28)

A 58 year old female with MM, planning for ASCT, admitted to our hospital on March 21, 2017.She was diagnosed MM (lambda), Durie-Salmon II A,ISS I, and underwent nine times chemotherapy with VCAD regimen (bortezomib, cyclophosphamide, adriacin and dexamethasone) in other hospital. Her other past medical history was negative. The patient’s physical examination was unremarkable. Blood cell count, renal/hepatic function, serum protein, serum κ and λ, immunoelectrophoresis, bone marrow examination, karyotype were all normal. She was evaluated to have complete remission. Electrocardiogram (ECG) showed heart rate of 56 beats per minute. Her 24 h dynamic electrocardiogram (Holter) suggested the average heart rate was 59 beats per minute. The patient was evaluated by sequential 2D Echocardiography, troponin I (Tpn-I), creatine kinase (CK) -MB and brain natriuretic peptide (BNP) which were all normal before ASCT. Peripheral blood stem cells were collected following cyclophosphamide (1.4 g × 3d) and recombinant human granulocyte colony-stimulating factor (rhG-CSF) mobilization (5.18 × 10^8^ mononuclear cells /kg, 2.19 × 10^6^ CD34^+^ cells /kg). On April 21, 2017, high dose melphalan at 200 mg/m^2^ in two divided doses on d-3 and d-2 was administered. Acute gastrointestinal reactions, including nausea, vomiting, diarrhea and oral mucositis were occurred on d + 2 and continued for about 10 days. On d + 6 she felt palpitation, ECG suggested atrial premature beats, then she was treated with oral metoprolol tartrate and ECG became normal. On d + 9, she felt palpitation again, ECG revealing paroxysmal AF and atrial flutter, then she was treated with metoprolol tartrate again, however, it didn’t improve her symptoms (Fig. 1a). For low blood pressure, pharmacological cardioversion was achieved using oral amiodarone 200 mg daily on d + 15 for 3 days. However, she developed chest tightness, shortness of breath, followed by ass attack on d + 18(Fig. 1b). Holter suggested AF with long RR interval and heart rate alternates fast and slow. Since ass attack occurred frequently, atropine and isoproterenol were promptly administered, but couldn’t control it. Therefore, a temporary heart pacemaker was placed on d + 19. Three days later, Holter suggested 1) paroxysmal AF and atrial flutter, lasted a few seconds. 2) paroxysmal sinus arrest occurred at the end of AF. Long intervals of RR with more than 2 s (13 times), and the longest RR was 4.1 s. 3) junctional escape rhythm and escape beat were observed sometimes. 4) ventricular pacing, ventricular intermittent pacing dysfunction. 5) frequent atrial premature (3917 times), partial two-triplet rhythm. 6) occasional ventricular premature (57 times) (Fig. 1c). She was diagnosed paroxysmal AF,atrial flutter,paroxysmal sinus arrest and cardiogenic syncope in sick sinus. On d + 28 because of sinus arrest and the long interval of RR, she was embedded in two-chamber permanent heart pacemaker (Fig. 1d). She received 2 years of lenalidomide maintenance therapy for MM. She is in good condition to this day.

## Discussion

The landscape of MM has changed dramatically over the last several years,with numerous new therapies and improved patient outcomes. ASCT is an effective therapy for transplant-eligible patients after induction therapy in newly diagnosed MM and relapsed patients who remain transplant eligible. Single-agent melphalan, at a dose of 200 mg/m^2^, is often used as a conditioning regimen for MM [1, 6].

The relationship between chemotherapy and arrhythmias has not been well established. The mechanism of AF and SVT after melphalan is not very clear. Increased age (> 60 years), higher baseline creatinine, larger left atrium size and previous cardiac comorbidities are noted risk factors for SVT, of which our patient hadn’t any risk factors [4]. The patient’s pre-melphalan evaluation of heart was unremarkable with performance status, left ventricular ejection fraction (55%), normal BNP, Tpn-I, CK-MB and Holter. Arrhythmia was reported as a side effect of many chemotherapeutic drugs. Guglin [3] reviewed published reports on chemotherapy-induced arrhythmias in English using the PubMed/Medline and OVID databases from 1950 to 2009 and found melphalan was associated with AF in 7~12% of cases, but it didn’t appear to cause ventricular tachycardia. In this case, multiple chemotherapy drugs were administered many times in the treatment of the patient include cyclophosphamide, anthracyclines and thalidomide, any of which could be associated with the cardiac toxicity [3, 7, 8]. However, she stopped chemotherapy 1 month before ASCT. Two days before ASCT, she just treated with high dose melphalan (200 mg/m^2^) as conditioning regimen. So, judging from the time of arrhythmia occurred, we highly speculated it should be a possible side effect of high dose melphalan.

The patients with MM always have immunoglobulin light chain amyloidosis. Abnormal serum-free light chains deposit in numerous organs, resulting in damage to the cardiac, hepatic, renal and nervous systems. Deposition of amyloid fibrils in the heart results in a restrictive cardiomyopathy, although amyloid deposition in the atria can result in atrial arrhythmias. Amyloid infiltration of the conduction system may lead to intraventricular conduction delay, as well as progressive conduction disease, heart block, ventricular arrhythmias and death [9]. The patient hadn’t any cardiac disease history. Before ASCT and after ASCT, we all examined BNP, Tpn-I, CK-MB which were all normal. The patient hadn’t congestive right-sided heart failure symptoms and echocardiographic features didn’t suggestive of amyloid deposition include the speckled appearance of myocardium and thickened interventricular septum. So, we didn’t find evidence of cardiac amyloidosis. However, we couldn’t completely exclude little light-chain amyloid depositing in conduction system but it was difficult to confirmed.

In addition, acute gastrointestinal reactions including loss of appetite, nausea, vomiting, diarrhea and oral mucositis occurred after high dose melphalan therapy and lasted for 10 days, which easily induced to abnormal electrolyte status. Hypokalemia was found after ASCT, but it was mild and recovered quickly. So we think this factor may be contribute to the arrhythmia, but not the major reason.

The manifestation of arrhythmia was tachy-brady syndrome of the patient. Radiofrequency ablation was the alternative choice for paroxysmal AF and atrial flutter, however, the platelets were very low at that time, heparin anticoagulation was needed during radiofrequency ablation, and the risk of bleeding was high, so it was impossible. She continued to have long interval of RR and frequent arrhythmia despite a temporary pacemaker implantation for 10 days, so it was advised by cardiologist to place permanent heart pacemaker.

MM is the third most common type of malignancy associated with cardiovascular diseases [10]. There are several contributing factors,such as chemotherapeutic medications, ASCT, cardiac amyloid deposition for the increased incidence of cardiac arrhythmias in patients with MM. We present this case due to the rarity and severity of sinus arrest secondary to high does melphalan and should monitor closely. With growing use of chemotherapy, cardiotoxicity is increasingly recognized as a common side effect. Cardiologists and hematologists should be involved in the whole course of treatment.

## Data Availability

Datasets for the manuscript can be available in word/excel format.

## Abbreviations

AF: Atrial fibrillation
ASCT: Autologous stem cell transplantation
BNP: Brain natriuretic peptide
CK: Creatine kinase
ECG: Electrocardiogram
Holter: 24 h dynamic electrocardiogram
LVEF: Left ventricular ejection fraction
MM: Multiple myeloma
rhG-CSF: Recombinant human granulocyte colony-stimulating factor
SVT: Supraventricular tachycardia
